# Creatine Supplementation for Patients with Inflammatory Bowel Diseases: A Scientific Rationale for a Clinical Trial

**DOI:** 10.3390/nu13051429

**Published:** 2021-04-23

**Authors:** Theo Wallimann, Caroline H. T. Hall, Sean P. Colgan, Louise E. Glover

**Affiliations:** 1 Department of Biology, ETH-Zurich, Emeritus, 8962 Bergdietikon, Switzerland; 2 Mucosal Inflammation Program and Department of Pediatrics, Division of Gastroenterology, Hepatology and Nutrition, Children’s Hospital Colorado, University of Colorado, 12700 E. 19th Ave, Aurora, CO 80045, USA; caroline.hall@childrenscolorado.org; 3 Mucosal Inflammation Program and Department of Medicine, Division of Gastroenterology and Hepatology, University of Colorado, 12700 E 19th Ave, Aurora, CO 80045, USA; sean.colgan@cuanschutz.edu; 4 Comparative Immunology Group, School of Biochemistry and Immunology, Trinity Biomedical Sciences Institute, Trinity College Dublin, D2 Dublin, Ireland

**Keywords:** pleiotropic effects of creatine (Cr) supplementation, inflammatory bowel diseases (IBD), ulcerative colitis, Crohn’s disease, creatine kinase (CK), phosphocreatine (PCr), creatine transporter (CrT), intestinal epithelial cell protection, intestinal tissue protection, creatine perfusion, organ transplantation, Adenosine mono-phosphate (AMP), activated protein kinase (AMPK), liver kinase B1 (LKB1), mitochondrial permeability transition pore (mPTP), reactive oxygen species (ROS), glucose transporter (GLUT)

## Abstract

Based on theoretical considerations, experimental data with cells in vitro, animal studies in vivo, as well as a single case pilot study with one colitis patient, a consolidated hypothesis can be put forward, stating that “oral supplementation with creatine monohydrate (Cr), a pleiotropic cellular energy precursor, is likely to be effective in inducing a favorable response and/or remission in patients with inflammatory bowel diseases (IBD), like ulcerative colitis and/or Crohn’s disease”. A current pilot clinical trial that incorporates the use of oral Cr at a dose of 2 × 7 g per day, over an initial period of 2 months in conjunction with ongoing therapies (NCT02463305) will be informative for the proposed larger, more long-term Cr supplementation study of 2 × 3–5 g of Cr per day for a time of 3–6 months. This strategy should be insightful to the potential for Cr in reducing or alleviating the symptoms of IBD. Supplementation with chemically pure Cr, a natural nutritional supplement, is well tolerated not only by healthy subjects, but also by patients with diverse neuromuscular diseases. If the outcome of such a clinical pilot study with Cr as monotherapy or in conjunction with metformin were positive, oral Cr supplementation could then be used in the future as potentially useful adjuvant therapeutic intervention for patients with IBD, preferably together with standard medication used for treating patients with chronic ulcerative colitis and/or Crohn’s disease.

## 1. Introduction

Inflammatory bowel disease (IBD), including ulcerative colitis (UC) and Crohn’s disease, are chronic and relapsing-remitting inflammatory disorders of the gastrointestinal tract that may develop in genetically susceptible individuals in response to unknown antigenic triggers. Although the etiology of IBD remains a conundrum, it seems definitive that multiple factors, such as genetic predisposition, environment, malfunction of the immune system and changes in the intestinal gut microbiota are involved in the onset and progression of IBD (for review see [1]). Since, in most cases, IBD cannot be cured completely, adjuvant therapies for IBD that may alleviate symptoms and thus improve quality of life parameters of afflicted patients, is a poorly characterized area of study [2].

Oral supplementation with chemically pure creatine (Cr) monohydrate, a natural nutritional supplement with pleiotropic beneficial influences, may fill such a gap in treatment options [3]. Cr may be a good candidate for an adjuvant treatment of IBD, since it has been shown to generally improve the energy state of cells, to enhance resilience of cells against several cell stressors, to modulate the immune system, to display anti-inflammatory influences and to dampen nociceptive pain, as will be elucidated below. 

Besides water, Cr is one of the most abundant single molecular compound in the human body, with a propensity of 120–140 g in a 70 kg person and a concentration of 40–50 mM in fast-twitch skeletal muscle. Cr monohydrate has become a very popular and the most effective ergogenic aid for athletic and recreational sports [4] and is used also as clinical adjuvant therapeutic intervention for patients with neuromuscular diseases [5]. Millions of persons worldwide consume Cr and, as one of the most intensively studied nutritional supplements, it is recommended as effective and safe by international sports societies [6] and national associations for food safety, such as EFSA and others. A dosage of 3–5 g of chemically pure Cr monohydrate per day as a long-term maintenance dose, without interruption needed, is generally recommended. For athletes in weight lifting and high-intensity performance disciplines, a short-term loading phase over 5–7 days with 20 g per day is recommended to quickly load the endogenous Cr pool in the muscles before switching to the above maintenance dosage [4]. Based on 30 years of practical experience with Cr supplementation and a very large number publications on this issue, involving athletes and regular people, as well as patients (children and elderly), oral supplementation with chemically pure Cr monohydrate if taken within the officially recommended dosages is safe for humans with no significant side effects (see the series of publications in this issue: https://www.mdpi.com/journal/nutrients/special_issues/creatine_supplementation, accessed on 2 March 2021, and in an earlier issue (https://link.springer.com/journal/726/48/8/page/1, accessed on 2 March 2021). As a matter of fact, Cr is necessary for optimal cell and body physiology, for genetic defects in either one of the two enzymes for endogenous Cr synthesis (AGAT or GAMT) or in the creatine transporter (CrT), the latter facilitating Cr uptake into target cells, lead to more or less severe Cr-deficiency syndromes in transgenic animal models, as well as in humans (for review see [7]).

### 1.1. The Phospho-Creatine Creatine Kinase System in Intestinal Epithelial Cells

**Figure 1:**
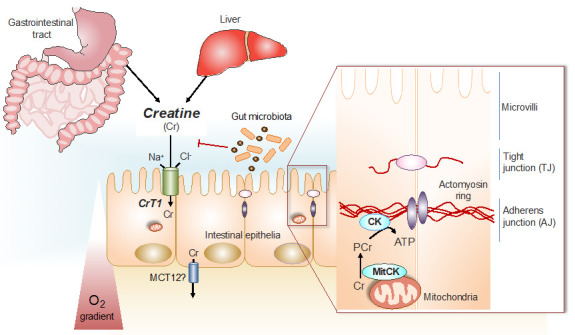
Cr/CK shuttle and the intestinal mucosal barrier. Cr is derived from dietary sources in the gastrointestinal tract, or by de novo synthesis primarily in the kidney and in the liver [12]. The Na^+^ and Cl^−^ dependent creatine transporter (CrT1), expressed in the apical membrane of intestinal epithelial cells, facilitates Cr uptake from the gut lumen [11,14]. Potential routes for Cr absorption into systemic circulation include paracellular movement by solvent drag transport, or via basolateral Cr transport by the monocarboxylate transporter 12 (MCT12) [15]. Gut microbiota express specific enzymes that can mediate Cr and creatinine (Crn) breakdown. In hypoxic intestinal epithelial cells, cytosolic CK localizes to apical adherens junctions in complex with the actomyosin cytoskeletal network, providing a conduit for rapid ATP generation during the energy-dependent processes of epithelial junction assembly and barrier restitution [16]. Adapted from [17].

The creatine kinase (CK)/phospho-creatine (PCr) system, with Cr as an energy precursor, plays a crucial physiological role for cells and tissues with high and fluctuating energy requirements, including skeletal, heart and smooth muscles, brain and nervous tissues, as well as other tissues and cells [3,8,9]. This also holds true for intestinal smooth muscle and intestinal epithelial cells, where cytosolic brain-type BB-CK and mitochondrial mtCK isoenzymes are prominently co-expressed [10] (see Figure 1). In addition, a specific creatine transporter (CrT1), belonging to the X-linked gene SLC6A8, as a member of a solute carrier family, is present in the apical cell membrane of intestinal epithelial cells [11]. By this electrogenic Na^+^- and Cl^−^-dependent Cr-cotransporter (CrT1), with a high affinity for Cr (Km for Cr of 30 µM), epithelial cells of the intestine take up Cr ingested with the diet, e.g., from meat and fish, as the most significant alimentary sources for Cr [12]. As CrT1 function is dependent on Na^+^, inhibition of the Na^+^/Ka^+^-ATPase, e.g., by the action of Lyn kinase, also inhibits Cr uptake into cells, as shown recently [13].

In humans, a significant proportion of the Cr taken-up via CrT1 into intestinal epithelial cells, is released into the blood stream by a basolateral monocarboxylate transporter, MCT12, that works as a novel facilitative CrT2 transporter [15]. From the blood, Cr is taken up by the target organs, such as skeletal and cardiac muscle, as well as neuronal tissues and other cells [12]. The uptake of Cr by intestinal epithelial cells, followed by trans-epithelial release into the blood stream leads to a systemic exposure of the body by Cr that is then taken up by those target organs, which depend on Cr, via their own CrT. Intestinal epithelial cells themselves also depend on the CK/PCr system for optimal physiological function. In these cells, PCr works in a similar way, as has been shown to be the case in other cells with high energy requirements [3], as an immediate high-energy buffer and as an energy transport vehicle to guarantee the maintenance of locally high PCr/ATP and ATP/ADP ratios in the vicinity of ATP-dependent processes, such as ion pumps and metabolite transporters [3,8,9], thus increasing the thermodynamic efficiency of intestinal epithelial cells [17] in a similar way as had been shown with other cells [3,8,10].

### 1.2. Creatine for Cytoprotection against Ischemia, Hypoxia, Oxidative Stress and Acidosis

Experimental and clinical data strongly support profound cell-protective properties of creatine in neuronal cells and tissues in vitro and in vivo [18,19,20]. Similar beneficial effects have also been observed with direct intra-venous phospho-creatine (PCr) injections against hypoxic cardiovascular stress [21]. Hypoxia also seems to play a key role in the pathogenesis of intestinal mucosal epithelial diseases [22], compromising cellular energy metabolism by lowering the cellular energy charge, i.e., the PCr/ATP ratio. Hypoxia renders cells more vulnerable to cellular stressors, such as ROS, inflammation and toxins [20,23,24]. In addition, hypoxia has been shown to diminish Cr uptake into cardiac cells [25]. Although the above positive influences of Cr on cell metabolism and cell integrity have largely been studied in other tissues and cells compared to in intestinal epithelial cells, the physiological workings of the CK/PCr system, the CrT and of creatine as such turned out to be very similar in the different cells and, thus, they are likely to also hold true for intestinal epithelial cells. Indeed, it was recently shown that hypoxia profoundly decreases levels of Cr, PCr and total available energy (PCr + ATP + (0.5 × ADP) in intestinal epithelial cells [26]. Thus, creatine supplementation is likely to normalize the intracellular Cr and PCr levels and thus also the energy charge, exemplified as the PCr/ATP ratio, in intestinal epithelial cells. At the same time, creatine supplementation is also highly cytoprotective against oxidative stress by ROS [23,27]. Creatine also leads to a significant increase in cell survival after hypoxic insult in vivo [19,20,28]. Recent work using transgenic mice that overexpress mtCK in cardiac muscle by only 25% over the normal level, shows that hearts from these mice functionally recover much better from ischemia reperfusion damage, with a significant reduction in cardiac infarct size [29]. This complements nicely with earlier data showing that transgenic mice overexpressing the creatine transporter (CrT) in heart and thus importing more creatine into this tissue, are also more resistant to ischemia reperfusion-related cardiac tissue damage. In addition, the extent of tissue damage is significantly lower in the mtCK overexpressing mice compared to normal control mice without elevated creatine concentrations in their heart muscle [30]. Given the cytoprotective role of creatine in the setting of hypoxia and ischemia referenced above and the evidence that hypoxia occurs in the setting of IBD [31] with associated intestinal cell barrier dysfunction [32], it follows that creatine is likely to provide a protective influence on the concomitant hypoxia observed in IBD.

Finally, as acidosis promotes lipid peroxidation and other manifestations of oxidant-mediated damage in various cell types, this condition is also relevant for intestinal epithelium. Acidosis, associated with inflammatory conditions, produces oxidative stress and amplifies these effects, e.g., at an acidotic pH, the response of the intestine to an oxidative insult is magnified [33]. In a rat model of chronic acidosis, creatine supplementation was shown to exert direct anti-oxidant properties by directly scavenging ROS and creatine abolished the chronic reduction in the expression levels of glucose transporter (GLUT2) [34]. Moreover, the administration of creatine under chronic acidosis led to functional strengthening of this jejunal acidotic phenotype, making the tissue more resistant to acidosis [34]. Thus, the beneficial influence and alleviation by creatine with respect to ischemic, oxidative and acidotic insults is certainly relevant for intestinal epithelial tissue, as well as for the entire intestine.

### 1.3. Creatine Stimulates Mitochondrial Respiration and Serves as an Anti-Apoptotic Effector

Mitochondrial creatine kinase (mtCK) and creatine stimulate mitochondrial respiration [35] and thus contribute significantly to maintain a healthy energy state of cells, especially under metabolic stress or toxic insults. Within this context, MtCK and creatine also play a crucial role in an early event of apoptosis; that is, in controlling the opening of the so-called mitochondrial permeability transition pore (mPTP) [36] that is sensitive to cyclosporine A. The addition of creatine to liver mitochondria from transgenic mice expressing mtCK in their livers, after a challenge by 40 mM calcium plus 5 mM atractyloside, prevents swelling of mitochondria and the release of apoptotic factors and reactive oxygen species (ROS) in a similar fashion as the bona fide anti-apoptotic agent cyclosporine A. In liver mitochondria from normal mice, which do not express mtCK in their liver, no effect of creatine on mitochondrial swelling could be seen, indicating that the action of mtCK, located in the mitochondrial inter-membrane space and present in all cells except for liver, is necessary together with creatine to prevent challenged mitochondria from swelling and mitochondrial permeability transition pore (mPTP) from opening [37]. Similar anti-apoptotic protection by creatine or phospho-creatine could be observed with intact cardiomyocytes [38], or with human umbilical vein endothelial cells that both were protected by creatine from lipopolysaccharide (LPS)-induced apoptosis [39]. This anti-apoptotic effect of creatine could also be demonstrated in vivo in hyper-cholesterolemic mice, where pravastatin-induced mitochondrial mPTP opening in skeletal muscles was minimized by creatine [40]. A significant factor for cell protection by creatine is mediated by the action of octameric mtCK that stabilizes mitochondrial contact sites and protects mitochondrial and cell integrity [41]. Thus, creatine, together with mtCK, regulate mitochondrial oxidative phosphorylation and exert a significant anti-apoptotic effect on a variety of cells by protecting them from different cytotoxic insults (for review [42]). In line with this notion, the anti-apoptotic effects and preservation of the function and structural integrity of mitochondria under metabolic stress was demonstrated directly in vivo in murine cardiac muscle [30]. Intestinal epithelial cell apoptosis significantly contributes to the development of ulcerative colitis and IBD in humans and mice, and therapies that target the inflammatory cytokine TNF, as well as the p53-upregulated modulator of apoptosis (PUMA) that are both upregulated in colitis tissues, have been found to inhibit apoptosis in intestinal epithelial cells and to promote mucosal healing [43]. Taking the evidence for the role of creatine in inhibition of apoptosis in other tissues and the finding that apoptosis contributes to disease activity in IBD [44], it is thus very reasonable to postulate that creatine would be anti-apoptotic in the mucosa of IBD patients as well.

### 1.4. Creatine as Anti-Inflammatory, Nociceptive and Immune Modulatory Compound

There is solid evidence in sports medicine, e.g., from ironman competitions, that creatine supplementation reduces the plasma levels of pro-inflammatory cytokines and prostaglandin E2 (PGE2) [45]. Very recent data have provided evidence that creatine may also have the potential to lower pain sensitivity associated with inflammation by antagonizing the acid-sensing ion channel (ASIC3) [46]. This effect is most likely based on the structural similarity of creatine, itself a guanidino compound, to other guanidino compound ligands of the ASIC3 pain receptor, such as GMQ and amiloride, that modulated this ion sensing channel [46]. Thus, one may expect from creatine supplementation, as an additional beneficial effect, an improvement of the abdominal pain associated with intestinal inflammation. This is an important determinant of quality of life for patients with IBD both in the setting of active inflammation as well as in IBD patients with irritable bowel syndrome symptoms in the absence of inflammation [47].

Immune cells themselves express CK and seem to depend on the CK/PCr system in a similar way to muscle and brain cells [3,10], as pointed out in a recent review [48]. For example, leucocytes express the CRT-1 transporter for the import of extracellular creatine [49]. Additionally, in macrophages, CK has a functional impact on these cells by supporting the formation of actin-based protrusions needed for macrophage motility and phagocytosis [50]. In addition, the uptake and accumulation of creatine into macrophages leads to the reprogramming and polarization of macrophages by modulating cellular responses to cytokines, such as IFN-γ and Il-4, thus enhancing the ability of macrophages to sense viral and bacterial antigens [51].

With regard to T cells, creatine kinase is involved in T cell development and activation [52] and creatine uptake regulates CD8 T cell immunity [53]. Thus, it seems obvious that the CK/PCr system has a profound impact both on the innate and adaptive immune response, exhibiting significant immune modulatory effects [54] and, therefore, it may be inferred that patients with IBD, who often suffer from intestinal infections, may benefit by creatine supplementation as a general activator of immune responses [54].

### 1.5. Creatine Affords Anti-Depressant Effects

There is growing evidence from human genetics, epidemiology, neuroimaging, as well as from animal studies that disruptions in brain energy metabolism, e.g., brain energy production, storage and utilization in the form of PCr and Cr [55] are implicated in the development and maintenance of depression, and that creatine has the potential to improve these disruptions in some depressive patients [56].

With respect to the clinical fact that many chronic colitis patients not only suffer from abdominal pain but also from depression, it is important to note that creatine has been shown to exert beneficial effects in the clinical management of depression, even in patients resistant to conventional anti-depression treatment [57,58]. Exciting new experiments with animal models show that creatine is able to afford its anti-depressant-like effects in a similar way as ketamine does [59,60]. Depression is a common finding in patients with chronic diseases, including those with IBD in whom depression significantly worsens their quality of life [61]. Thus, it will certainly be worthwhile to test the anti-depressant effects of creatine supplementation in our cohort of chronic colitis patients.

## 2. Scientific Rationale Specifically for Intestinal Tissue

### 2.1. HIF Controls Creatine Kinase (CK) Expression and CK Together with Creatine Are Involved in the Energetics of Mucosal Barrier Regulation

**Figure 2:**
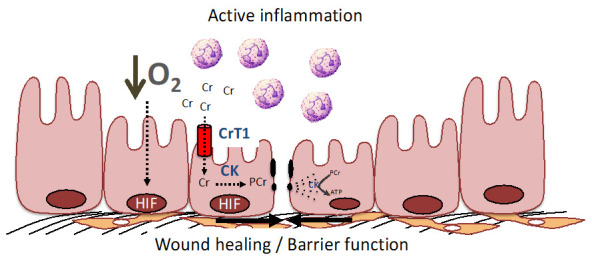
The PCr/Cr shuttle promotes mucosal barrier and wound healing by improving cellular energetics and by stabilization of the adherence junctions. During active inflammation, such as that seen in IBD, low O_2_ levels result in the stabilization of HIF and resultant induction of creatine kinase (CK) isoenzymes and the Cr transporter (CrT1) within intestinal epithelial cells (see [16,62,63] for further details). CK localizes to epithelial junctions that are stabilized by interactions with the actin cytoskeleton. In response to epithelial disruption during inflammation, large amounts of ATP are necessary to accommodate the demand for cytoskeletal reorganization, including the acto-myosin ATPase at epithelial cellular junctions. Under such conditions, CK and CrT1 coordinately promote wound healing and barrier function by generating ATP from PCr to efficiently promote homeostasis.

Fully charged cellular energy batteries are a prerequisite for optimal body function not only for muscle and brain cells, but also for intestinal smooth muscle and epithelial cells. Long-term decay or failure of cellular energetics, e.g., by chronic ischemia, inflammation and progressive dysfunction of mitochondria, as well as deterioration of mucosal barrier functions, are important aspects of IBD that are accompanied by a state of chronic inflammation [17]. Mucosal surfaces of the lower gastrointestinal tract are subject to pronounced fluctuations in oxygen (O_2_) tension, particularly during inflammation (Figure 2). As an adaptive response to hypoxia, the hypoxia-induced transcription factors (HIF-1 and HIIF-2) become stabilized [62]. An unbiased analysis of HIF target genes identified creatine kinases (both cytosolic BB-CK and mtCK) and the major Cr transporter SLC6A8 that are all coordinately regulated by HIF [16]. Further analysis revealed that cytosolic BB-CK is expressed in a HIF-2 dependent manner and that this enzyme localizes to apical intestinal epithelium cell adherence junctions, where it is critically involved in the ATP-dependent junction assembly, epithelial integrity and mucosal barrier function (see Figure 1). This same study revealed that tissue transcripts from 30 IBD patients (including both Crohn’s disease and ulcerative colitis) showed a marked reduction in the expression of all three isoforms of CK compared to non-IBD controls. In light of this observation, it is notable that the interaction of epithelial junctions with the actin cytoskeleton is a significant energy sink within the mucosa. Energy deficiencies associated with IBD, including those associated with microbial dysbiosis, likely contribute to barrier dysfunction during active inflammation [63].

### 2.2. Creatine Supplementation Regulates the Energy Balance of Intestinal Epithelial Cells, Epithelial Integrity and Barrier Function

**Figure 3:**
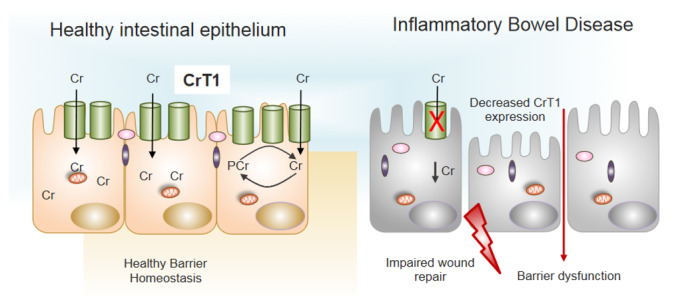
Decreased expression of CrT1 in IBD promotes barrier dysfunction. Normal expression of CrT1 on the apical surface of intestinal epithelia (left panel) results in adequate supplies of Cr via dietary sources to promote healthy barrier function and intestinal homeostasis. Patients with IBD express lower levels of CrT1 (right panel) and disrupt the Cr-PCr energy shuttle to the extent that wound healing potential and barrier are dysfunctional (see [14,16] for further details).

Patients with IBD present with intestinal barrier dysfunction that is likely related to disturbed cellular energetics and dysbiosis. Since the CK/PCr system, as well as the creatine transporter (CrT1) are involved in a plethora of processes that are important for cellular energetics [3,8], also in intestinal epithelial cells [16,17,62] it is interesting that the investigation of mucosal biopsied from 30 patients with Crohn’s disease and 27 patients with ulcerative colitis both showed lower expression levels of CrT1, which might contribute to the reduced barrier function of intestinal epithelium [14] (see Figure 3). Notably, in intestinal epithelial cells (IECs), CrT1 localized specifically around tight junctions and knockdown or overexpression of CrT1 in these cells corroborated the idea that CrT1, besides regulating the intracellular creatine concentration in IECs, was also modulating epithelial barrier formation and wound healing [14]. In CrT1 knockdown IECs—that is, in the absence of adequate creatine transport—these cells transformed to a stressed, glycolysis-predominant energy metabolism, resulting in leaky tight junctions and mislocalization of actin and tight junction proteins [14]. Despite the significant impacts of CrT1 loss, proliferation was not altered in CrT1 knockdown intestinal epithelial cells [14]. It is noteworthy that metabolomic analysis has revealed that the actin cytoskeleton demands nearly 20% of total available energy within the epithelium [26]. Taken together, these data support the fact that CrT1, together with CK, phosphocreatine (PCr) and creatine, regulates the energy balance of IECs and enforces the structural and functional integrity of the tight-junction-actin cytoskeleton. These are excellent arguments that speak for a clinical trial, using creatine supplementation directly on patients with IBD.

### 2.3. Creatine Supplementation Maintains Intestinal Epithelial Energy Homeostasis and Protects against Colitis in Animal Models

Oral creatine supplementation in mice with experimentally induced colitis markedly ameliorated both disease severity and inflammation in TNBS and DSS mouse colitis models [16]. Furthermore, as an indicator for pathology, mucosal CK expression was lowered in patients with ulcerative colitis and Crohn’s disease. Thus, a role for HIF-regulated CK expression in intestinal epithelial homeostasis was established and this revealed a fundamental link between cellular bioenergetics and mucosal barrier [16,64]; see schematic representation of CK function for maintaining intestinal mucosal barrier function in [17]. This model (see Figure 1) is representative of the well-documented PCr shuttling function from mitochondria, via mitochondrial mtCK to intracellular sites of ATP consumption, where cytosolic CK is specifically localized for in-situ ATP-regeneration [3,8,9,12].

### 2.4. Creatine-Loading Preserves Intestinal Barrier Function during Intestine Organ Preservation by Static Cold Storage

Very recent data demonstrate that, in two rodent models, a single flush of intestines with intraluminal preservation solution supplemented with 50 mM Cr significantly improved intestinal barrier function and electrophysiology, reflecting superior mucosal integrity after 10 h of cold storage, in comparison to the placebo group without Cr [65]. Permeability and trans-epithelia resistance measurements remained at fresh tissue values and oxidative injury was controlled in the Cr group by the preferential utilization of glutathione [65]. Thus, Cr supplementation of intestinal tissue improved graft quality, among others, by preserving the cellular energy state in this tissue, which is reflected in a greater PCr energy charge of 324% in Cr treated versus control intestinal tissue. The significant improvement in cellular energy state, reflected by an increased PCr/ATP, as well as ATP/AMP ratios in the Cr group, was most likely responsible for alleviating tissue damage due to the ischemic storage of intestine. Thus, augmenting the cellular energy reserves by Cr supplementation of intestinal tissue led to improved tissue integrity and physiological function, such that a fully energized state is facilitated upon the reperfusion of transplanted intestinal tissue [65]. Similar cell-protecting effects against ischemia or ischemia-reperfusion tissue damage were already described for cardiac muscle and brain tissues [3,7,19,20,21,23,55]. The above data show that perfusion of intestinal tissue of the mouse, before transplantation, has a tissue protective effect and improves the results of intestine organ transplantation. If these data are confirmed with human patients, the perfusion of intestinal tissue with Cr could become a standard procedure in intestinal tissue transplantation.

### 2.5. A Genetic Screen with Mice Susceptible for Colitis Reveals a Link to Creatine Metabolism

The data presented above are supported by a full-fletched genetic study involving the screening of 36′530 third generation germ-line mutant mice, derived from N-ethyl-N-nitroso-urea-mutagenized grandsires for abnormalities in intestinal homeostasis and abnormalities after oral administration of dextran sodium sulfate (DSS) to induce colitis [66]. The team around Dr. Bruce Beutler, Nobelist in Physiology, 2011, of Texas Southwestern Medical Center in Dallas, identified, among 27 mice susceptible to the colitis phenotype, one mutant mouse that was strongly correlated with a missense mutation in one of the two enzymes that are important for endogenous creatine biosynthesis; that is, arginine-glycine-aminotransferase (AGAT) catalyzing the rate-limiting first step of creatine synthesis, mainly taking place in the kidney [12]. The intestinal epithelium of the AGAT mutant mice displayed significantly increased cell death and decreased proliferation during DSS treatment, compared to control mice under the same challenge. Then, the supplementation of homozygous AGAT mutants with exogenous creatine ameliorated and significantly improved the histological parameters and the colitis phenotype of these mice, respectively [66]. These findings establish an in vivo requirement for the rapid replenishment of cytoplasmic ATP by the CK/PCr-system within colonic epithelial cells for the maintenance of the mucosal barrier after injury [66].

### 2.6. Involvement of AMPK Activation to Restore Adherence Junction Assembly in Intestinal Epithelium

Adenosine monophosphate-activated protein kinase (AMPK), an evolutionarily conserved serine/threonine protein kinase, plays a central role in the maintenance of the cellular energy balance [67]. AMPK works as a cellular energy sensor, responding to cellular energy stress situation, e.g., oxidative stress, ischemia and anoxia, as indicated by an elevated AMP/ATP ratio. Under such conditions, specifically arising in pathological cases of IBD, AMPK is activated both by allosteric binding of AMP [68], as well as by upstream kinases. For example, AMPK is phosphorylated and activated by liver kinase B1 (LKB1) [69], a protein kinase that plays a conserved role in epithelial polarity and regulation [70]. Several lines of experimental evidence point towards an important role of AMPK in the regulation of epithelial adherence junction assembly and disassembly and demonstrates an intriguing link between cellular energy status and adherence junction function [71]. For example, the activation and phosphorylation of AMPK increases during calcium-induced adherence junction assembly and this increase depends on the activity of LKB1. Conversely, a kinase-dead mutant of AMPK inhibits adherence junction assembly and barrier function, as measured by the localization of specific adherence junction proteins and by trans-epithelial resistance, respectively [71]. In fact, metformin a widely prescribed, clinically safe anti-diabetes drug and well-known activator of AMPK [72] promotes the expression and assembly of adherence junctions via an AMPK-dependent way [73]. This is corroborated by the fact that a reduced risk of IBD is consistently observed in patients with type 2 diabetes mellitus, who have been treated with metformin [74].

Therefore, not only CK and the PCr/Cr energy support system, but also AMPK, as a regulator of cellular energetics, play a direct role in the structural and functional integrity or adherence junctions and thus for epithelial barrier function. These data would argue that one could combine and thus enforce oral creatine supplementation with low-dose metformin treatment as adjuvant therapy for IBD (see below).

### 2.7. Creatine Supplementation in One Single Case of Crohn’s Disease Improved Both Symptomatic and Endoscopic Characteristics of Ulcerative Colitis

Fully in line with these pioneering data is a first single case study with a 33-year-old patient with a two-year history of Crohn’s ileitis, who responded very well to creatine supplementation (1.5 g per day, given as monotherapy for a time period of 6 months) with both symptomatic and endoscopic improvement in disease activity [75]. Specifically, before creatine supplementation, the colonoscopy of the patient showed large ulcers of 0.5–2.0 cm in diameter with >30% ulcerative surface, 50–70% affected surface and no narrowing (SDS-CD:7), whereas after creatine supplementation, the same patient presented with aphthous ulcers <0.5 cm in diameter, <10% ulcerated surface, <50% affected surface and no-narrowing (SES-CD:3) (see Figures 1–3 in [75]).

## 3. Proposal, Methodology and Clinical End-Points

Based on the theoretical concept of CK function and experimental evidence from the DSS colitis mouse models, as well as on the single case clinical trial, plus the extensive genetic screen that revealed a correlation of colitis to a failure in creatine metabolism (mutated AGAT), a pilot clinical trial is ongoing. Indeed, a randomized, placebo-controlled clinical pilot trial with creatine supplementation (2 × 7 g of creatine monohydrate per day, ingested for 8 weeks, 6 patients per arm) is underway in 12 patients, aged 18–70, with mild to moderate ulcerative colitis at the University of Colorado (NCT02463305). Note that this study is not a monotherapy trial and patients taking mesalamine or thiopurines will be allowed concomitant use of these drugs during the trial. The primary outcome is improvement in the endoscopic score as an assessment of mucosal inflammation from biopsy samples obtained before and after the 8-week treatment course. Multiple secondary outcomes include intestinal permeability, as measured by urinary saccharide excretion testing, symptom severity, colonic inflammatory biomarkers, CK and Cr levels from biopsies and blood, intestinal microbiome as well as clinical remission status. This ongoing trial should give insight into the tolerability, safety and efficacy of creatine monohydrate for patients with ulcerative colitis.

(A) Creatine monotherapy trial: In addition to the current study mentioned above, we believe that there is utility in conducting a detailed investigation into the long-term effects of creatine supplementation in IBD which expands on the above study. Such a Cr monotherapy trial could include a careful evaluation of 40 patients treated with placebo (control group) and 40 patients treated with creatine monotherapy (2 × 3–5 g of Cr per day for a time of 3–6 months (verum group) and should be carried out by using standard clinical questionnaires for ulcerative colitis and Crohn’s disease, (e.g., IBDQ). Intestinal inflammation should be quantitatively assessed using established inflammation biomarkers (e.g., fecal calprotectin, serum C-reactive protein) and intestinal permeability should be quantified using urinary saccharide excretion. Finally, patients should undergo pre- and post-treatment coloscopy, currently the gold standard of assessment for mucosal inflammation. Patients with diagnosed colorectal cancers will be excluded from the study. The reason for this precaution is that, in an animal model with orthotopically implanted colorectal cancer cell lines, very high dosage of external Cr fed to these cancer mice promoted cancer metastasis, resulting in a higher incidence of liver metastases compare to control animals without externally added Cr [76]. However, the amounts of orally fed Cr to the cancer mice corresponded to a calculated amount of 50–150 g of Cr/day for a human of 70 kg body weight. On the other hand, in accordance with earlier studies [48,77,78], the same authors found that Cr instead had an anti-proliferative effect on primary tumor growth. As a matter of fact, Cr has convincingly been shown to represent an important metabolic regulator controlling anti-tumor T cell immunity [53], which also represents a key player in the etiology of IBD’s. Based on these results, Cr supplementation has been proposed as a valid strategy in cancer protection and/or management [79], as well as to prevent cancer-related loss of weight and muscle mass [80]. Questionnaires, measurement of clinical parameters, as well as coloscopy, should be performed at the beginning and after three and six months of the clinical study.

(B) Creatine treatment in combination with metformin: As stated above, metformin has shown a great potential in experimental cell and animal models of IBD [71,72,73] and offered interesting retrospective insights from patients with type 2 diabetes, who took metformin [74]. Therefore, a combination treatment with 2 × 3–5 g Cr per day plus a low dose of metformin (1 × 0.5–1.0 g/day) may be suggested, for the two are likely to work synergistically and may provide better clinical results compared to creatine monotherapy alone. Since the safety profile of both, creatine and metformin, is excellent, such a clinical study should be straight-forward, considering that millions of people take either creatine or metformin for other purposes.

## 4. Anticipated Outcome

The anticipated outcome will be a proof of concept from the ongoing Cr adjuvant clinical pilot study in mild to moderate ulcerative colitis. It is also proposed that a Cr monotherapy pilot using 2 × 3–5 g of chemically pure creatine monohydrate per day, given for 3–6 months, either as a monotherapy or in combination with low dose metformin (1 × 0.5–1.0 g per day) would potentially be able to improve several quality-of-life parameters of patients with chronic colitis, e.g., improvement in muscle strength, mobility and vital functions, with less fatigue and depression. Most importantly, one would expect a possibly significant reduction or even alleviation of colitis symptoms, such as abdominal pain, clinical symptoms and inflammation of the colon, as judged by coloscopy. Almost certainly, since IBDs are multifactorial diseases with phenotypical and genetic subtypes, Cr is unlikely solving all the problems associated with these serious diseases, but if Cr should indeed turn out to be helpful in inducing such favorable responses or even the remission of the ulcerative pathology in colitis patients, an inclusion of creatine supplementation, either alone or in combination with metformin, as standard adjuvant therapeutic intervention for ulcerative colitis and/or Crohn’s disease, could be envisaged, preferably together with established medical treatment options. As a prerequisite for this, however, a multicenter study with significant patient numbers will be needed. Excluding the animal experiments and one human case, described above, no other clinical study with Cr supplementation has been performed with IBD patients so far. Therefore, it is to be expected that, based on the results of the proposed studies, either with creatine and metformin alone or in combination, some potentially unique information concerning potentially beneficial effects of Cr on colitis and Crohn’s disease should be revealed by such a study as a clinical endpoint.

## 5. Conclusions

Based on the available experimental data and the scientific rationale, it seems appropriate and timely to propose a full-fletched clinical trial with oral creatine monohydrate supplementation alone or in combination with metformin on patients suffering from IBD. Depending on the outcome of the results proposed herein with Cr supplementation and/or metformin alone or in combination, such treatments could in the future become a standard adjuvant therapeutic intervention for ulcerative colitis and/or Crohn’s disease that could be combined with established medical treatments for these pathological situations, hopefully for the benefit of IBD patients.

## Data Availability

Data is contained within the article.
